# Facilitating MR-Guided Adaptive Proton Therapy in Children Using Deep Learning-Based Synthetic CT

**DOI:** 10.14338/IJPT-20-00099.1

**Published:** 2021-06-25

**Authors:** Chuang Wang, Jinsoo Uh, Thomas E. Merchant, Chia-ho Hua, Sahaja Acharya

**Affiliations:** Department of Radiation Oncology, St Jude Children's Research Hospital, Memphis, TN, USA

**Keywords:** synthetic CT, adaptive proton therapy, cycle GAN, deep learning

## Abstract

**Purpose:**

To determine whether self-attention cycle-generative adversarial networks (cycle-GANs), a novel deep-learning method, can generate accurate synthetic computed tomography (sCT) to facilitate adaptive proton therapy in children with brain tumors.

**Materials and Methods:**

Both CT and T1-weighted magnetic resonance imaging (MRI) of 125 children (ages 1-20 years) with brain tumors were included in the training dataset. A model introducing a self-attention mechanism into the conventional cycle-GAN was created to enhance tissue interfaces and reduce noise. The test dataset consisted of 7 patients (ages 2-14 years) who underwent adaptive planning because of changes in anatomy discovered on MRI during proton therapy. The MRI during proton therapy-based sCT was compared with replanning CT (ground truth).

**Results:**

The Hounsfield unit-mean absolute error was significantly reduced with self-attention cycle-GAN, as compared with conventional cycle-GAN (65.3 ± 13.9 versus 88.9 ± 19.3, *P* < .01). The average 3-dimensional gamma passing rates (2%/2 mm criteria) for the original plan on the anatomy of the day and for the adapted plan were high (97.6% ± 1.2% and 98.9 ± 0.9%, respectively) when using sCT generated by self-attention cycle-GAN. The mean absolute differences in clinical target volume (CTV) receiving 95% of the prescription dose and 80% distal falloff along the beam axis were 1.1% ± 0.8% and 1.1 ± 0.9 mm, respectively. Areas of greatest dose difference were distal to the CTV and corresponded to shifts in distal falloff. Plan adaptation was appropriately triggered in all test patients when using sCT.

**Conclusion:**

The novel cycle-GAN model with self-attention outperforms conventional cycle-GAN for children with brain tumors. Encouraging dosimetric results suggest that sCT generation can be used to identify patients who would benefit from adaptive replanning.

## Introduction

The enhanced-dose sculpting capability of pencil-beam scanning proton therapy is associated with increased sensitivity to anatomic changes. Although 27% of pediatric patients demonstrate anatomic changes during therapy, current treatment planning methods do not effectively account for anatomic variation [1]. This could potentially result in suboptimal delivered plans, defined as inadequate coverage of tumor or increased dose to healthy structures. Furthermore, the process of adapting plans to changing anatomy is resource intensive: it often requires repeat computed tomography (CT) simulation, particularly if there is a shift in brain tissue or resolution of postsurgical fluid adjacent to the resection cavity. For young children, a repeat CT simulation increases their exposure to both anesthesia and ionizing radiation. Therefore, we proposed using synthetic CT (sCT), derived from an offline on-treatment magnetic resonance imaging (MRI), acquired routinely during proton therapy (MRI_tx_) to (1) calculate the delivered dose for the anatomy of the day and flag cases that would benefit from adaptation, and (2) implement MR-only adaptive proton planning.

Deep learning, specifically a cycle-generative adversarial network (cycle-GAN), is a more recent approach to generating sCT from MR images that overcomes challenges inherent to atlas-based and voxel-based methods [2–5]. With cycle-GAN, standard MRI sequences can be used without the restriction of paired CT/MR datasets for training, which minimizes the effect of image misalignment. However, most of the studies using deep-learning approaches have focused on adults, have been limited by small training datasets, and have not assessed the application of sCT to adaptive proton therapy [6–8]. Although adult brain tumors are commonly located in the cerebral cortex, pediatric brain tumors are commonly located in the posterior fossa, extending into the upper cervical cord or suprasellar region, adjacent to the sphenoid and nasal sinuses. Therefore, proton therapy beam paths commonly used in pediatric patients are different from those used in adults. Pediatric tumors, such as craniopharyngiomas and optic pathway gliomas, are located adjacent to bone-air interfaces, which can be challenging to accurately synthetize from an MRI [6, 7]. Although beam angles are chosen to minimize traversal through sinus regions, for tumor extending into the sella turcica, bone-air interfaces cannot be avoided (**Supplemental Figure S1**). Another challenge relevant to the pediatric population is that tumors, such as ependymomas and medulloblastomas, can extend inferiorly into the spinal canal (**Supplemental Figure S1**). Inaccuracies in vertebral bone synthesis within the beam path can significantly affect the dose distribution. Furthermore, pediatric patients are also more likely to present with hydrocephalus and require shunting, which causes artifacts on MRI. Therefore, a pediatric-specific model, trained on a pediatric dataset, is warranted. Only one deep-learning–based sCT study has focused on pediatric patients, to our knowledge; however, it did not apply sCT in an adaptive context or address challenges unique to the pediatric population [9].

We hypothesized that by introducing a novel, self-attention mechanism to cycle-GAN, we could improve boundary delineation at air-bone-tissue interfaces and enhance local details, relative to the results obtained with conventional cycle-GANs for pediatric brain tumors. We also hypothesized that proton therapy plans calculated on an sCT generated by a self-attention cycle-GAN would appropriately trigger plan adaptation.

## Materials and Methods

### Adaptive Concept Overview

We have previously described our offline MRI-guided adaptive proton therapy workflow [1]. During proton therapy, patients undergo offline MRI_tx_ to detect anatomic changes that affect delivery accuracy. When a change in target volume or healthy tissue is detected on MRI_tx_, a repeat CT is usually acquired to calculate the original plan on the anatomy of the day and to reoptimize the plan (**Supplemental Figure S2**). Reasons for acquiring a repeat CT include a shift in the target volume or healthy brain tissue or resolution of postoperative fluid adjacent to the surgical cavity.

### Patient Data for Model Training and Testing

The training dataset comprised 125 pairs of simulation CT and same-day T1-weighted (T1W) MRI scans from 125 patients who underwent both scans between 2016 and 2019, excluding the test patients. Thirty-one (25%) out of 125 training patients had a shunt. None of the patients in the training dataset required adaptive planning. The testing dataset included 7 patients who required adaptive planning because of anatomic changes during proton therapy and who had both T1W MRI_tx_ and replanning CT available. Three of the 7 test patients had shunts (**Supplemental Table S1**). The maximum time interval between the T1W MRI_tx_ and the replanning CT was 3 days. The median time interval between the initial simulation CT and the replanning CT was 27 ± 11 days. Reasons for plan adaptation included target volume shift (n = 1), target volume enlargement (n = 2), target volume reduction (n = 3), and change in tissue heterogeneity (n = 1). The median age of the patients in the training and testing datasets was 8.3 years (range, 1-20 years) and 6.4 years (range, 2-14 years), respectively. It is important to note that 4 of the 7 patients had tumors in the suprasellar region, adjacent to nasal and sphenoid sinuses, and 1 patient had a target volume in the fourth ventricle, extending inferiorly into the spinal canal. All histologies represented in the testing dataset were also represented in the training dataset. This study was approved by the institutional review board at St Jude Children's Research Hospital (19-0322).

### Image Acquisition

All CT studies were acquired in treatment positions on a Spectral CT (IQon, Philips Healthcare, Cleveland, Ohio) with 120 kVp, 0.36 pitch, 0.625-mm collimation, autoexposure control, and a 50 cm field of view. The images were reconstructed with an iterative algorithm (iDose4, level 2) to yield a pixel size of 0.98 by 0.98 mm^2^ and a slice thickness of 1 to 2 mm. The T1W MRI_tx_ images were acquired in treatment position using a Turbo Field Echo sequence on 1.5T or 3T MRI systems (Ingenia, Philips Healthcare, Gainesville, Florida). To replicate treatment position, flexible-loop coils and a head-positioning overlay board were used to accommodate face masks and other immobilization devices, as described previously [10].

### Image Preprocessing

The T1W MR scans were rigidly registered to the corresponding CT using the FMRIB (FMRIB Analysis Group, Oxford, UK) linear image registration tool [11] to homogenize image size. For each patient, a binary mask was generated, which included the entire cranium and upper cervical spine until the C2-C3 junction. The registered MR and CT were symmetrically cropped to 256 by 256 pixels in anterior-posterior and left-right directions to remove background and keep the region of interest in the center of the image (**Supplemental Figure S3**). Patient anatomy was kept within the region of interest. To reduce the intensity inhomogeneity of MR images across slices, slice-based normalization was applied to scale the pixel intensity of cropped T1W MR_tx_ to within −1 and 1. Volume-based normalization was applied to CT images to give the same intensity range of (−1 and 1).

### Self-attention Cycle-GAN

**Figure 1.**
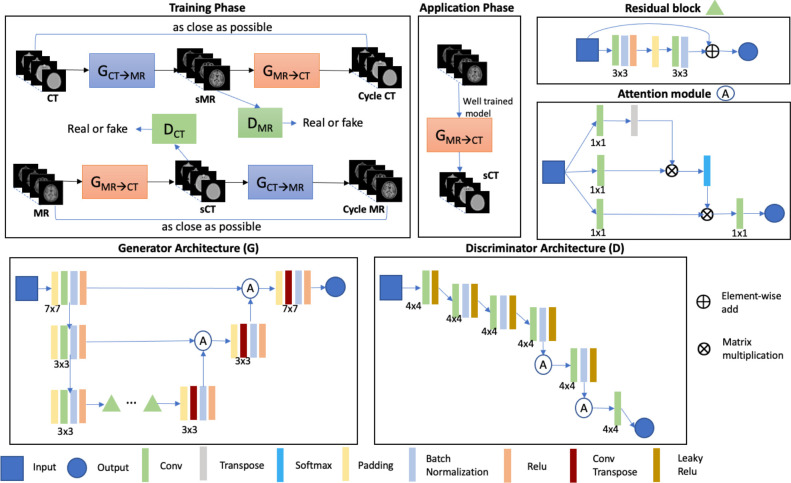
Schematic of the self-attention cycle-GAN for MR-based sCT generation. Abbreviations: Conv, convolutional layer; Conv Transpose, convolutional transpose layer; CT, computed tomography; cycle-GAN, cycle-generative adversarial network; D, discriminator; DCT, CT discriminator; DMR, MR discriminator; G, generator; GCTs→MR, generator of sMR from CT; GMR→CT, generator of sCT from MRI; MR, magnetic resonance; MRI, magnetic resonance imaging; Relu, rectified linear unit layer; sCT, synthetic computed tomography; sMR, synthetic MRI.

Convolutional neural network– or conventional GAN-based methods rely on accurate spatial registration between the paired CT and MR images to generate sCTs. However, local misalignment by 2 to 5 mm can occur, even after spatial registration [12]. One of the advantages of the cycle-GAN-based method is to allow unpaired image-to-image training, which minimizes the training error from imperfect image alignment. Furthermore, cycle-GAN [13] incorporates an inverse transformation that converts CT to MR and MR back to CT, which generates optimal pseudo pairs with a one-to-one mapping. To improve the performance further, a self-attention mechanism [14] was introduced into the generator and discriminator of the conventional cycle-GAN to enhance the local details and reduce the blurry boundary commonly present at tissue interfaces. The self-attention module was applied to the last 2 layers of both discriminator and generator, based on a previously published model [14]. The self-attention module models long-range, multilevel dependencies across image regions and facilitates processing of high-level features to improve performance. To avoid overfitting, we limited the self-attention module to the last 2 layers. **Figure 1** shows the architecture of the self-attention cycle-GAN. The self-attention module builds relationships between separated spatial regions, such that the boundaries between such regions are enhanced. The input features from the previous hidden layer *x* ∈ ℝ^*c × w × h*^ are transformed into 3 feature spaces *f*, *g*, and *h* as *f*(*x*) = *W_f_x*, *g*(*x*) = *W_g_x*, and *h*(*x*) = *W_h_x*. The attention map can be defined as:


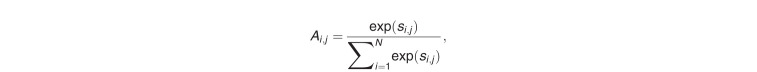

where *s_i_*_,_*_j_* = *f*(*x_i_*)*^T^g*(*x_j_*). *A_i_*_,_*_j_* builds the relationship between the *i*th location and the *j*th region. The attention module is placed in the last 2 layers in the generator and the discriminator to build the long-range dependencies in the images after refining each spatial location. Nine residual blocks [15] were used in the generator to handle the complicated transformation from a large variation in head shape and size, considering the wide age range (1-20 years) in this cohort.

### Training and Implementation

The model was implemented by pyTorch (https://pytorch.org) and trained on a single NVIDIA (Santa Clara, California) Tesla P100 GPU (graphics processing unit) computing processor with 16 GB of memory. The Adam (adaptive moment estimation) algorithm [16] was applied to optimize the learning with an initial learning rate of 10^−4^ and a momentum term of 0.5 without applying a dropout. The maximum number of training epochs was 200 with the early stopping rule [17]. A batch size of 2 was used for training in our study. Model training required a week of computations and took 30 seconds to generate MR-based sCT for each test patient.

### Evaluation

To determine the efficacy of the self-attention module, the cycle-GAN models with and without self-attention were trained and tested on the same dataset. For the latter model, the architecture adopted in **Figure 1** was kept the same, except that the self-attention module was removed. The performance of the 2 models was compared visually and in a quantitative manner with the image quality metrics described below.

For both cycle-GAN models, the generated sCT was compared with the real CT (ground truth) on image quality and dosimetric accuracy for 7 test patients. Image-quality metrics included peak signal-to-noise ratio (PSNR), structural similarity index (SSIM), and voxel-based mean absolute error (MAE) in Hounsfield units (HU) were calculated inside the binary mask. Only pixels within the binary mask were included for image-quality comparison, thereby excluding the couch and immobilization device. A commercial treatment planning system (Eclipse 15.1, Varian Medical System, Inc, Palo Alto, California) was used to design the original and adapted treatment plans on initial CT and replanning CT, respectively. For dosimetric evaluation, background images outside the head on planning CT were first added to the sCT to restore the immobilization device and couch information. The original plan was applied to sCTs and replanning CTs to calculate dose distribution on the anatomy of the day, defined as the delivered plan on sCT (dsCT) and the delivered plan on replanning CT (drCT), respectively. The adapted plan was also applied to the sCT (asCT) and replanning CT (arCT). Each individual dsCT and asCT was dosimetrically compared with the corresponding drCT and arCT (ground truth). To evaluate the dose differences, dose-volume histogram analysis and 3-dimensional (3D) gamma analysis with 2%/2 mm criteria and a 10% dose threshold was performed. To investigate the difference in proton range between dsCT and drCT, as well as asCT and arCT, the R80 (80% distal falloff in the beam axis) for each beam was calculated. Dice coefficients of selected isodose lines (95%, 80%, 50%, and 20%) were also calculated to compare the similarity of the isodose distributions and the plan conformality.

### Statistical Analysis

The MAEs between different models were compared with paired *t* tests. *P* ≤ 0.05 was considered significant. Stata (College Station, Texas) software (version 16SE) was used for all analyses.

## Results

The HU MAE was significantly reduced with self-attention cycle-GAN, as compared with conventional cycle-GAN (65.3 ± 13.9 versus 88.9 ± 19.3, *P* < .01) (**Table 1**). There were smaller improvements in mean PSNR (27.7 ± 2.2 versus 28.5 ± 2.2, *P* = .52) and mean SSIM (0.88 ± 0.04 versus 0.90 ± 0.03, *P* = .56) with self-attention. Patient 2 demonstrated the worst performance (MAE, 92.1 HU) because of the presence of a shunt, which was associated with a very large MR artifact (**Supplemental Figure S4**). When analyzing different tissue compartments with cycle-GAN with self-attention, MAE was greatest in bone (102 ± 12 HU), followed by air (53 ± 7 HU) and brain parenchyma (28 ± 12 HU) (**Table 2**). Dice coefficients for air and bone were > 0.8.

**Table 1.** Image-quality comparisons of synthetic computed tomography (sCT) generated by cycle-generative adversarial network (cycle-GAN) with and without self-attention in 7 test patents.

**Patient No.**	**Self-attention cycle-GAN**			**Cycle-GAN**		
	**PSNR, dB**	**SSIM**	**MAE, HU^a^**	**PSNR, dB**	**SSIM**	**MAE, HU**
1	31.4	0.93	52.3 ± 38	30.2	0.92	65.7 ± 35
2	25.7	0.85	92.1 ± 48	24.2	0.81	126.2 ± 49
3	26.5	0.87	53.4 ± 47	26.4	0.86	73.7 ± 46
4	27.5	0.89	57.2 ± 50	26.5	0.88	83.3 ± 35
5	29.6	0.92	61.5 ± 43	29.0	0.92	91.2 ± 32
6	31.2	0.92	71.9 ± 38	30.1	0.92	86.6 ± 32
7	28.1	0.90	68.7 ± 45	27.7	0.88	95.7 ± 39
Mean^b^	28.5 ± 2.2	0.90 ± 0.03	65.3 ± 13.9	27.7 ± 2.2	0.88 ± 0.04	88.9 ± 19.3

**Abbreviations:** PSNR, peak signal-to-noise ratio; SSIM, structural similarity index; MAE, voxel-based mean absolute error; HU, Hounsfield unit.

a Mean absolute error ± SD on a voxel level.

b Mean ± SD of patients 1 to 7.

**Table 2.** Tissue compartment comparison between computed tomography (CT) and synthetic CT (sCT) generated by cycle-generative adversarial network (cycle-GAN) with and without self-attention in 7 test patents.

**Patient No.**	**Self-attention cycle-GAN**					**Cycle-GAN**				
	**Air, HU < −800**		**Bone, HU > 200)**		**Brain**	**Air, HU < −800)**		**Bone, HU > 200)**		**Brain**
	**MAE,^a^ HU**	**DSC**	**MAE, HU**	**DSC**	**MAE, HU**	**MAE, HU**	**DSC**	**MAE, HU**	**DSC**	**MAE, HU**
1	52 ± 35	0.85	95 ± 101	0.76	30 ± 16	61 ± 41	0.83	137 ± 153	0.74	47 ± 19
2	61 ± 59	0.83	111 ± 97	0.79	32 ± 13	64 ± 57	0.82	172 ± 123	0.77	52 ± 21
3	61 ± 47	0.81	128 ± 131	0.80	41 ± 19	69 ± 63	0.78	181 ± 117	0.77	47 ± 25
4	49 ± 53	0.89	101 ± 89	0.90	12 ± 7	53 ± 56	0.87	157 ± 134	0.85	31 ± 18
5	48 ± 55	0.90	110 ± 107	0.81	43 ± 21	46 ± 49	0.91	174 ± 176	0.79	58 ± 35
6	52 ± 61	0.85	101 ± 95	0.77	24 ± 11	57 ± 59	0.84	165 ± 171	0.77	34 ± 23
7	41 ± 45	0.92	95 ± 93	0.87	15 ± 12	48 ± 60	0.90	143 ± 128	0.85	26 ± 17
Mean ± SD^b^	52 ± 7	0.86 ± 0.03	107 ± 12	0.81 ± 0.05	28 ± 12	57 ± 9	0.85 ± 0.05	161 ± 16	0.79 ± 0.04	42 ± 12

**Abbreviations:** MAE, voxel-based mean absolute error; HU, Hounsfield unit; DSC, dice similarity coefficient.

a Mean absolute error ± SD on a voxel level.

b Mean ± SD of patients 1 to 7.

**Figure 2.**
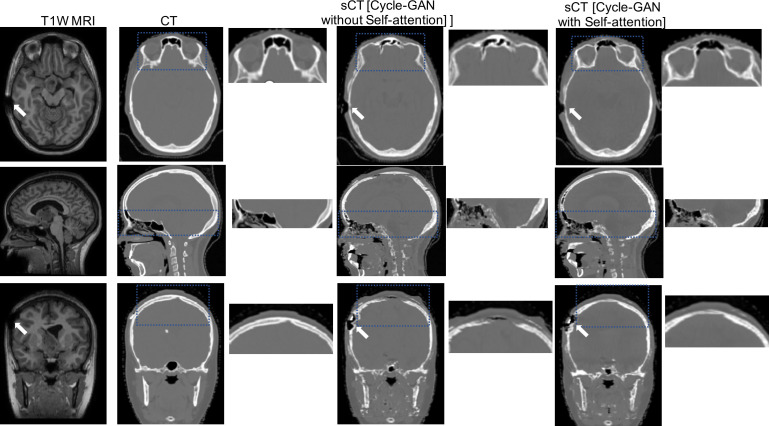
From left to right: T1W MRI_tx_, replanning CT images and its zoom in view, sCT from cycle-GAN without self-attention and its zoom in view, and sCT from cycle-GAN with self-attention and its zoom in view. Abbreviations: CT, computed tomography; cycle-GAN, cycle-generative adversarial network; MRI, magnetic resonance imaging; MRI_tx_, acquired routinely during proton therapy; sCT, synthetic computed tomography; T1W, T1-weighted.

A detailed, qualitative comparison of real CTs to sCTs generated by cycle-GAN, with and without self-attention, is presented for patient 3 (**Figure 2**). Addition of the self-attention mechanism improved the bony definition of the orbits (axial view) and the base of the skull and sinuses (sagittal view) and reduced the blurry boundary at the superior extent of the cranium (coronal view). The MR artifact associated with the shunt was perpetuated in the sCT for both cycle-GAN models (white arrows in **Figure 2**).

**Figure 3.**
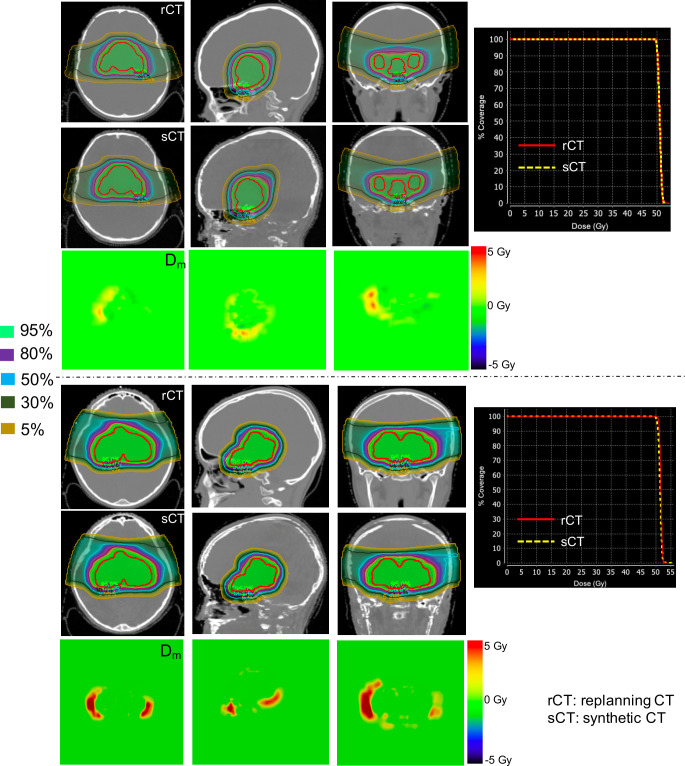
Differences in dose map and CTV DVH between sCT and replanning CT for the adapted plan for patient 5 (top panel) and patient 7 (bottom panel). Red contour = CTV. The dose map was generated for the total prescribed dose. Abbreviations: CT, computed tomography; CTV, CT cerebral venography; DVH, dose-volume histogram; sCT, synthetic computed tomography.

The dsCTs and asCTs were compared to the drCTs and arCTs for 7 test patients (**Table 3**). The 3D gamma passing rates, with 2%/2 mm criteria and a 10% dose threshold were 97.5% ± 1.1% between drCTs and dsCTs and 98.9% ± 0.9% between arCTs and asCTs. Across all 14 plans, the mean absolute difference in CT volume (CTV) receiving 95% of the prescription dose (D_95_), CTV receiving 95% of prescription volume (V_95_), and the R80 were 0.4% ± 0.2%, 1.1% ± 0.8%, and 1.1 ± 0.9 mm, respectively. Patient 6 had the largest differences in R80, and that was attributable to errors in synthetizing cervical bone adjacent to the target volume and inaccurate synthesis of bone within the target volume (**Supplemental Figure S4**). The dose conformality between plans calculated on sCTs, as compared to replanning CTs was similar, as indicated by the high dice coefficients for all selected isodose lines (**Supplemental Table S2**). The beam angles for all test patients are provided in **Supplemental Table S3**. The best and the worst 3D gamma passing rates when comparing arCTs to asCTs were demonstrated by patients 5 (**Figure 3**, top panel) and 7 (**Figure 3**, bottom panel), respectively. Although both of those patients had a suprasellar tumor located adjacent to the nasal cavity and sphenoid sinus, the patient-7 tumor tracked along the optic canal and was more than double the size of the patient-5 tumor (114.3 cm^3^ versus 52.2 cm^3^). The differences in dose were fairly small within the CTV. However, for patient 7, a larger dose difference was observed distal to the CTV, which reflected a shift (2.9 mm) in the distal falloff of one beam (**Table 3**).

**Table 3.** Dosimetric comparison of delivered (dsCT) and adapted plans (asCT) on synthetic computed tomography (sCT) and delivered replanning (drCT) and adapted replanning (arCT) computed tomography (CT).

**Patient No.**	**Comparison between drCT and dsCT**					**Comparison between arCT and asCT**				
	**Gamma passing rate, %**	**ΔD_95_, %**	**ΔV_95_, percnt;**	**ΔR80, mm (along beam angle 1)**	**ΔR80, mm (along beam angle 2)**	**Gamma passing rate, %**	**ΔD_95_, %**	**ΔV_95_, %**	**ΔR80, mm (along beam angle 1)**	**ΔR80, mm (along beam angle 2)**
1^a^	98.1	0.2	0.8	0.6	−0.7	98.8	0.6	0	0.7	−0.7
2	97.2	0.1	0	−0.3	−0.6	99.8	0.2	0.2	−2.1	−1.9
3	97.3	0.6	0.7	−0.1	1.9	99.4	0.6	1.5	0	2.1
4	96.5	0.1	0.9	−0.8	2.4	98.7	0.5	2.9	−0.7	−1.1
5	99.6	0.3	0.9	−0.4	−0.1	99.9	0.2	0.9	−0.2	1.0
6	96.5	0.4	1.4	−1.3	2.6	97.8	0.4	2.4	−0.3	3.3
7	97.2	0.4	0.9	1.7	−2.3	97.6	0.9	1.2	0.7	−2.9
Absolute mean ± SD	97.5 ± 1.1	0.3 ± 0.2	0.8 ± 0.4	0.7 ± 0.6	1.5 ± 1.0	98.9 ± 0.9	0.5 ± 0.2	1.3 ± 1.1	0.7 ± 0.7	1.9 ± 1.0

**Abbreviations:** Δ, absolute difference; D_95_, receiving 95% of the prescription dose; V_95_, receiving 95% of the prescription dose; R80, 80% distal falloff in the beam axis.

Note: The difference in D_95_, D_99_, V_95_, and R80 was calculated by subtracting the value for sCT from that for replanning CT.

a Patient 1 was treated with 3 beams, ΔR80 between delivered plan and dsCT, and between adapted plan and asCT along the third beam is 0 mm and −0.8 mm, respectively.

To determine whether sCT can detect dose differences that will appropriately trigger treatment with an adapted plan, we compared the difference between the delivered and adapted plans on sCTs and replanning CTs for the 7 test patients. The dose-volume parameters used to trigger adaptation included CTV V_95_ and brainstem V_95_ (**Supplemental Table S4**). Clinically, a difference of ≥ 5% in these dose-volume parameters has been used to determine whether or not to treat with an adapted plan. In all 7 test patients, adaptation was appropriately triggered when using the sCT to calculate differences between delivered and adapted plans (**Supplemental Table S4**).

## Discussion

We have developed a novel self-attention cycle-GAN model that outperforms conventional cycle-GAN in generating accurate MR-based sCTs for children with brain tumors. We also demonstrated proof-of-concept that sCTs based on the self-attention cycle-GAN can detect dose differences that will appropriately trigger plan adaptation. This is the first study, to our knowledge, to focus on a clinically relevant pediatric population, which poses unique challenges when compared with the adult population because of tumor locations adjacent to air-bone interfaces and along the spinal canal as well as the common presence of shunts.

Our method resulted in an MAE of 65 HU, which is comparable to that in recently published deep-learning studies in adults, for which MAEs on the order of 89 to 47 HU were reported [3, 6–8, 18]. None of the aforementioned studies evaluated sCTs in the context of adaptive proton therapy. The single deep-learning study in pediatric brain population reported a MAE of 61 HU [9]. There may be several reasons why the MAE in our cohort is higher than that reported for adult studies. First, in our cohort, higher MAEs were observed in patients with MR artifacts secondary to shunts. Shunts are common in pediatric patients with brain tumors. Twenty-five percent of patients in our training dataset and 29% of patients in our testing dataset had a shunt. Second, we used MR imaging from both 3T and 1.5T, which may have negatively affected our results. One way to address that problem would be to homogenize MR images in preprocessing stage before the generation of the synthetic CT. Third, there is no standard field-of-view with which to calculate MAEs in the literature. That means that some studies will only use the cranium with the inferior border at the foramen magnum, and other studies will include the upper cervical cord. Including the cervical vertebral bodies will increase the MAE because the MAE is greatest in bone. In our study, the inferior border was at the C2-C3 junction.

Although sCTs can be used to calculate delivered dose on anatomy of the day and flag cases for reoptimization, further work needs to be done before sCT is used for pediatric MR-only proton planning. Differences in R80 between sCTs and replanning CTs were not negligible and might be clinically meaningful if the distal falloff is within an organ at risk, such as the optic chiasm, particularly because the relative biologic effectiveness increases sharply at the distal end of the Bragg peak [19]. Therefore, caution should be used when relying on sCTs for beams that range out into organs at risk. Caution should also be used when evaluating tumors that extend along the bony orbital canal or cervical spine because inaccuracies in bone synthesis can result in 2- to 3-mm differences in the R80. Given that any model could potentially underperform under certain circumstances, future work should focus on developing a metric to predict proton therapy dose accuracy of an sCT when a “gold standard” comparison is not available. Developing such a metric would be critical to the successful clinical implementation of sCT for MR-only proton planning.

Our study had several limitations. Our testing dataset was small because we were interested in testing the ability of the model to function in an adaptive workflow and, therefore, we limited the testing dataset to patients who required plan adaptation during proton therapy. We did not exclude patients with shunts because we wanted to ensure our results would be generalizable to the pediatric population. Given that our testing cohort was small, our results should be interpreted as hypothesis-generating and should be validated in a larger cohort.

In conclusion, we have presented a novel method for generating MR-based sCTs for children with brain tumors that incorporates a self-attention mechanism into the conventional cycle-GAN. Compared with cycle-GAN without self-attention, our pediatric-specific model is associated with a significantly lower MAE and can reliably trigger plan adaptation.
